# The Nose

**Published:** 1890-04

**Authors:** 


### THE NOSE.

The nose acts like a custom-house officer to the system. It is highly sensitive to the odor of the most poisonous substances. It readily detects hemlock, henbane, monk's-hood, and the plants containing prussic acid ; it recognizes the fetid smells of drains, and warns us not to inhale the polluted air. The nose is so sensitive that it distinguishes air containing the two hundred thousandth part of a grain of the otto of rose, or the fifteen millionth part of a grain of musk. It tells us in the morning that our bedrooms are impure, and catches the fragrance of the morning air, and conveys to us the invitations of the flowers to go forth into the fields and inhale their sweet breath. To be lead by the nose has hitherto been used as a phrase of reproach ; but to have a good nose, and to follow its guidance, is one of the safest and shortest ways to the enjoyment of health.

